# Genetic Approach for the Fast Discovery of Phenazine Producing Bacteria

**DOI:** 10.3390/md9050772

**Published:** 2011-05-09

**Authors:** Imke Schneemann, Jutta Wiese, Anna Lena Kunz, Johannes F. Imhoff

**Affiliations:** Kieler Wirkstoff-Zentrum (KiWiZ) am Leibniz-Institut für Meereswissenschaften (IFM-GEOMAR), Am Kiel-Kanal 44, 24106 Kiel, Germany; E-Mails: ischneemann@ifm-geomar.de (I.S.); jwiese@ifm-geomar.de (J.W.); akunz@ifm-geomar.de (A.L.K.)

**Keywords:** phenazine, *Actinobacteria*, oligonucleotides, HPLC-UV/MS

## Abstract

A fast and efficient approach was established to identify bacteria possessing the potential to biosynthesize phenazines, which are of special interest regarding their antimicrobial activities. Sequences of *phz*E genes, which are part of the phenazine biosynthetic pathway, were used to design one universal primer system and to analyze the ability of bacteria to produce phenazine. Diverse bacteria from different marine habitats and belonging to six major phylogenetic lines were investigated. Bacteria exhibiting *phz*E gene fragments affiliated to *Firmicutes*, *Alpha-* and *Gammaproteobacteria*, and *Actinobacteria*. Thus, these are the first primers for amplifying gene fragments from *Firmicutes* and *Alphaproteobacteria*. The genetic potential for phenazine production was shown for four type strains belonging to the genera *Streptomyces* and *Pseudomonas* as well as for 13 environmental isolates from marine habitats. For the first time, the genetic ability of phenazine biosynthesis was verified by analyzing the metabolite pattern of all PCR-positive strains via HPLC-UV/MS. Phenazine production was demonstrated for the type strains known to produce endophenazines, 2-hydroxy-phenazine, phenazine-1-carboxylic acid, phenazine-1,6-dicarboxylic acid, and chlororaphin as well as for members of marine *Actinobacteria*. Interestingly, a number of unidentified phenazines possibly represent new phenazine structures.

## 1. Introduction

Nature is a profitable source of pharmaceutically active substances covering the whole range of biological activities such as antimicrobial, antitumoral, antiparasitic or immunosuppressive [1,2]. Various natural products or natural product-derived compounds have been approved as drugs or are undergoing clinical evaluation and registration [2,3]. Because the rediscovery rate of already known substances in standard screening approaches is high, new strategies are urgently needed. Genetic approaches for the detection of secondary metabolite pathways are promising tools for the selection of biosynthetically talented microorganisms. So far, main targets in this respect were genes encoding for polyketide synthases (PKSs) or non-ribosomal peptide synthetases (NRPSs) [4,5]. By focusing on polyketides and non-ribosomal peptides other interesting compound classes such as phenazines have been neglected so far. Phenazines of natural or synthetic origin turned out to be good drug candidates and therefore are promising secondary metabolites [6]. They are heterocyclic, nitrogenous compounds that are substituted at different sites of the core ring system and therefore display a wide range of structural derivatives and biological activities. More than 100 biologically active (antibacterial, antifungal, antiviral, antitumor) phenazines from natural origin are known to date, synthesized mainly by *Pseudomonas* and *Streptomyces* species [7]. Phenazines produced by the root-colonizing *Pseudomonas fluorescens* 2–79 and *Pseudomonas aureofaciens* 30–84 are good examples for congeners with promising biological activity, in this case inhibiting several fungal plant pathogens [8]. An economically successful phenazine is clofazimine (Lamprene®, Novartis), first described in 1957 which was established as an antileprosy agent and exerts anti-inflammatory properties in controlling *erythema nodosum leprosum* reactions (Official FDA Drug label) [6] as well as other antimicrobial activities [9–11]. Clofazimine is a synthetic phenazine analogue belonging to the riminophenazines, a group of compounds which was originally discovered in lichens [12,13]. Another phenazine, bis(phenazine-1-carboxamide), acts as a potent cytotoxin and represents an interesting class of dual topoisomerase I/II directed anticancer drugs [14].

**Figure 1.**
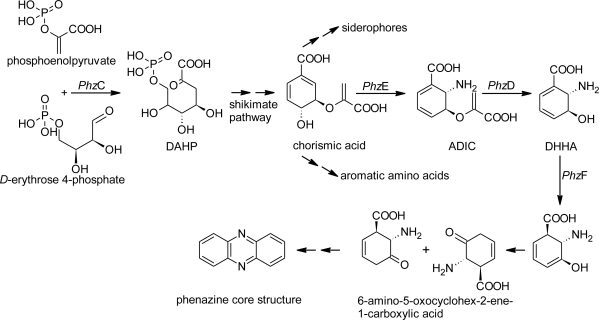
Schematic representation of the phenazine biosynthesis pathway.

Although principal parts of the phenazine biosynthesis (Figure 1) and their genetics are known [15,16], there are still gaps in our knowledge and the phenazine pathway is still hypothetical [17]. The function and gene products of *phz*C, *phz*E and *phz*D have been experimentally proven. These enzymes catalyze the conversion into 3-deoxy-d-*arabino*-heptulosonic acid 7-phosphate (DAHP), 2-amino-2-deoxyisochorismic acid (ADIC) and *trans*-2,3-dihydro-3-hydroxyanthranilic acid (DHHA) [17–20]. The phenazine core structure is synthesized subsequently by condensation of two molecules of 6-amino-5-oxocyclohex-2-ene-1-carboxylic acid [17]. Presumably, *Phz*F is responsible for the isomerization of DHHA [18]. By now, the function of the enzymes *Phz*A, *Phz*B and *Phz*G are almost enlightened [19] and their role in the condensation and rearrangement reactions to form PCA is demonstrated [17]. According to this information, the design of appropriate oligonucleotide probes and primers was possible. Mavrodi *et al.* (2010) [21] quite recently designed four different pairs of oligonucleotides on the basis of the *phz*F gene of the phenazine biosynthesis and investigated the diversity of phenazine producers mainly in soil-dwelling and plant-associated bacteria.

In particular marine bacteria have attracted our interest to search for promising natural products [1,22–26]. Therefore, we have investigated the possible application of a genetic approach for the detection of new marine isolates using *phz*E gene fragments as markers for the ability to produce bioactive phenazines. In contrast to Mavrodi *et al.* (2010) [21], we wanted to design a more universal primer system able to study a wide range of bacterial groups including actinomycetes and pseudomonades, in order to detect genes of phenazine production in unidentified new isolates without prior performance of a phylogenetic classification.

## 2. Results and Discussion

The high diversity of microbes and their manifold biosynthetic capabilities offer a great potential for novel, bioactive secondary products. Genetic approaches such as the application of primer systems for NRPS, PKS or halogenases are efficient methods to select appropriate strains for further analyses [27,28]. The screening of bacteria for the presence of phenazine genes significantly adds to these selection filters. We report here on a rapid universal genetic system for the discovery of bacteria that are able to produce phenazines. The suitability of this PCR based screening approach for the identification of phenazine producing bacteria was demonstrated. A gene fragment indispensable for phenazine biosynthesis (*phz*E) served as a template for primer generation. As positive controls the type strain of *S. cinnamonensis*, and three subspecies of *Pseudomonas chlororaphis*, which are all well known producers of phenazines, were included in this study. For control strains several phenazine biosynthesis gene sequences were available at the NCBI (e.g., AM384985, HM594285, AF007801). Altogether 168 bacterial strains (including the four reference organisms) were examined for the presence of *phz*E. The PCR analysis was complemented by a chemical investigation in order to demonstrate the expression of the biosynthesis genes and to confirm the production of phenazines as proof of concept.

### 2.1. Design of Oligonucleotides to Search for phzE Phenazine Gene Fragments

**Figure 2.**
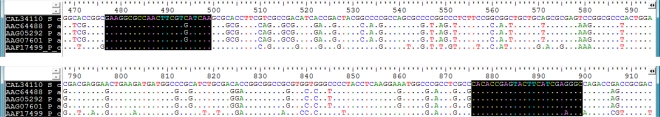
Alignment of known *phz*E gene sequences. Marked blocks served as the basis for primer construction.

Former investigations were able to associate different steps of the phenazine biosynthetic pathway with the corresponding genes, e.g., *phz*C, *phz*D, *phz*E and *phz*F. The transformation from chorismate to 2-amino-2-deoxyisochorismic acid (ADIC) is necessary for the formation of the core structure of phenazines and is catalyzed by the enzyme *Phz*E. Thus, *Phz*E is a key enzyme in phenazine biosynthesis and the corresponding gene *phz*E is suitable for primer design. Sequences from the phenazine biosynthetic pathway for *Alpha*-, *Beta*- and *Gammaproteobacteria, Actinomycetes* and *Firmicutes* are available at the homepage of the National Centre for Biotechnology Information (NCBI) and known from literature [29,30]. To ensure the inclusion of only true phenazine sequences, oligonucleotide primers were constructed only from those genes known to be involved in the biosynthesis of corresponding chemical substances. Two conserved sites occurred within the alignment of *phz*E sequences (Figure 2), which had a distance to each other to produce fragments of an appropriate length. The degenerated primers *phz*Ef (5′-GAA GGC GCC AAC TTC GTY ATC AA-3′) and *phz*Er (5′-GCC YTC GAT GAA GTA CTC GGT GTG-3′) were designed to amplify a highly conserved stretch of the *phz*E gene of approximately 450 bp. The comparison of the oligonucleotide sequences from designed *phz*Ef and *phz*Er primers with known phenazine genes verified this stretch as highly specific for *phz*E genes. Because the basic phenazine gene cluster including the *phz*E gene is highly conserved and derivatization of the basic phenazine structure are made at a later stage in the biosynthesis, the constructed *phz*E primers are expected to detect genes of a large variety of different phenazine structures and are appropriate to search for unknown bacteria producing novel phenazines.

### 2.2. Screening for phzE Gene Fragments with the Constructed Primers

**Figure 3.**
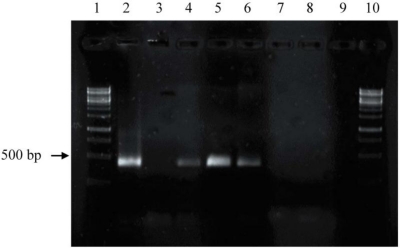
*Phz*E gene fragment amplification of selected strains. Lane 1 and 10: 1 kb DNA-ladder; 2: positive control *Pseudomonas chlororaphis* subsp. *chlororaphis* DSM 50083^T^; 3: negative control (without template DNA); 4, 5, and 6: isolates H253, HB117, and LB129, respectively, exhibiting *phz*E fragments; 7, 8, and 9: isolates HB290, HB147, and LB164, respectively, exhibiting nor *phz*E fragments.

Genes belonging to the phenazine biosynthetic pathway were present in approximately 10% of the bacterial strains analyzed. PCR results of 13 (8%) out of 164 bacterial strains and four reference organisms were positive in regard to the presence of *phz*E gene fragments (Figure 3, Tables 1 and 2). The investigated bacteria comprised different bacterial phyla, namely *Actinobacteria* (76), *Bacteroidetes* (2), *Firmicutes* (28) and *Proteobacteria* (62) (Table 1).

Corresponding gene fragments were detected in 11 strains of *Actinobacteria*, one strain of *Firmicutes* and two strains of the *Alphaproteobacteria*. All sequences were similar to known *phz*E gene sequences in a range from 65% similarity (*phz*E of strain LB151 to *phz*E of *P. chlororaphis*, AAF17499) to 95% similarity (*phz*E of strain AB108 to *phz*E of gene from *S. cinnamonensis*, CAL34110) (Table 2). Regarding the environmental isolates none of the strains within the *Bacteroidetes, Beta*- as well as *Gammaproteobacteria* could be shown to contain *phz*E in PCR amplification. This was unexpected, because among the 36 gammaproteobacterial isolates 18 *Pseudomonas* strains were examined and our PCR approach was performed with primer sequences largely based on sequences from *Pseudomonas* strains known as producers of phenazines [16,31,32]. The suitability of our primer set to detect phenazine genes in *Pseudomonas* species was further demonstrated by performing a database search that matched perfectly several phenazine genes, e.g., *P. chlororaphis* (L48339), *Pseudomonas sp.* M18 (FJ494909), *P. aeruginosa* (FM209186, CP000744, CP000438, AE004091, AF005404). Anyhow, a study based on *phz*F sequences exhibited a hit ratio of 100% including 51 *Pseudomonas* strains [21]. Therefore, all 18 pseudomonads from our study exhibiting negative results using *phz*E primers were subjected to a genetic approach with *phz*F primers. While the PCR-amplification of *phz*E and *phz*F gene fragments of the control type strains was positive (Table 2), amplification of the investigated isolates failed. As an additional control experiment, crude extracts of six *Pseudomonas* strains were analyzed by HPLC-UV/MS. Because of the distinctive properties of phenazine UV-absorption spectra the presence of phenazine metabolites was out of question. Additionally, for another study all natural products from two of the investigated *Pseudomonas* strains were isolated and chemically identified. No phenazines were detected. We conclude that the *Pseudomonas* strains analyzed in this study lack genes for phenazine production and are unable to produce phenazines. In agreement with this, the only known marine phenazine producing *Pseudomonas* species is *P. aeruginosa* [33–35], synthesizing almost always pyocyanin. In contrast, different marine streptomycetes are known for production of variable phenazine structures [9]. *Streptomyces* strains in this study are the most productive group as well. While *Brevibacterium*, *Bacillus* and *Pelagibacter* were known as marine phenazine producers [36–38], this is the first time that representatives of the genera *Micromonospora*, *Kiloniella* and *Pseudovibrio* were identified as marine phenazine producers as well.

### 2.3. Detection of Phenazines in the phz*E* Positive Strains

**Figure 4.**
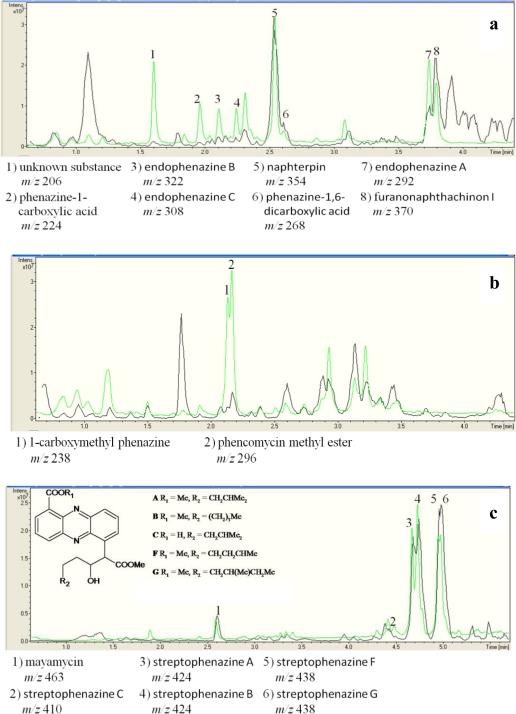
**(a)** UV/MS-chromatogram (black line: MS; green line: UV at 250 nm) of a 17 day-old GYM agar plate of strain *Streptomyces cinnamonensis* DSM 1042^T^. Endophenazine A-C, phenazine-1-carboxylic acid, phenazine-1,6-dicarboxylic acid and an unknown phenazine substance were detected as well as naphterpin and furanonaphthachinon I; **(b)** UV/MS-chromatogram (black line: MS; green line: UV at 250 nm) of a 17 day-old GYM agar plate of *Streptomyces* strain LB129. 1-carboxymethyl phenazine and phencomycin methyl ester were detected; **(c)** UV/MS-chromatogram (black line: MS; green line: UV at 250 nm) of a 17 day-old GYM agar plate of strain HB202. Different streptophenazines and the aromatic polyketide mayamycin were detected.

**Figure 5.**
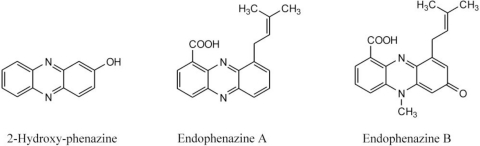
Structures of selected phenazines produced by *Pseudomonas chlororaphis* subsp. *chlororaphis* DSM 50083^T^ and *Streptomyces cinnamonensis* DSM 1042^T^.

To demonstrate the synthesis of phenazines in all *phz*E positive strains, cultures of these strains were extracted and analyzed by HPLC-UV/MS analyses. 14 out of 17 of these strains were able to produce one or more substances with molecular masses and UV-spectra similar to known phenazines (Table 3, Figure 4a–c). In *S. cinnamonensis* DSM 1042^T^ the production of endophenazines A–C (Figure 5) and phenazine-1,6-dicarboxylic acid [15] could be demonstrated (Figure 3a). The metabolite chlororaphin was discovered from *Pseudomonas chlororaphis* subsp. *chlororaphis* DSM 50083^T^. 2-hydroxy-phenazine (Figure 5) and phenazine-1-carboxylic acid were produced by *Pseudomonas chlororaphis* subsp. *aureofaciens* DSM 6698^T^ and *Pseudomonas chlororaphis* subsp. *aurantiaca* DSM 19603^T^. In addition, the presence of senacarcin A (strain *Streptomyces* sp. HB117), saphenyl ester D, aestivophoenin C and a derivative thereof (strains *Streptomyces* sp. HB122 and HB291) as well as phencomycin methyl ester and 1-carboxymethyl phenazine from strain *Streptomyces* sp. LB129 (Figure 3b) were identified.

All environmental isolates producing phenazines (6%) were marine *Streptomyces* sp. or *Micromonospora* sp. strains. Most of these strains produced both known phenazines and phenazines which did not show any accordance to a database entry. In total, 22 known phenazines were identified. In the case of strain *Streptomyces* sp. HB202 (Figure 4c), the production of streptophenazines A-H was verified using NMR spectroscopic analyses [39]. The large number of *Streptomyces* strains containing *phz*E genes is in good agreement with previous reports describing streptomycetes as a rich source for phenazines [9,15,39,40].

In nine of the culture extracts a total of 13 different substances showed typical phenazine like UV-absorption spectra, but gave no hit in the databases concerning UV and mass data. This indicates the presence of unidentified and possibly new natural phenazine products which warrant further investigation.

For some of the identified phenazines interesting biological activities were reported. Senacarcin A is known for its activity against Gram-positive bacteria and tumor cell lines [45] and aestivophoenin C has antioxidative activity and acts as a neuronal cell protecting substance [44]. Interesting bioactivities of phenazines are also known from the marine *Streptomyces* sp. strain HB202, which produced several streptophenazines with activity against Gram-positive bacteria [39].

We expect that investigation of other so far unidentified phenazines from marine *Actinobacteria* is a remunerative challenge. Interestingly, phenazines were not detected in culture extracts of *phz*E positive strains of *Alphaproteobacteria* and *Firmicutes*. Though, all bacteria containing a *phz*E phenazine gene fragment have the capability to synthesize the phenazine core structure, proof of gene fragments from a biosynthetic pathway does not give evidence of the integrity of corresponding gene cluster. Additionally, the expression of a gene cluster under conditions used is not warranted. Therefore, it is most likely that the cultivation conditions used were not appropriate for the production of some of the phenazines and have to be modified for the selected strains by our genetic approach in further studies.

## 3. Experimental Section

### 3.1. Bacterial Strains and Their Phylogenetic Affiliation

166 bacterial strains used in this study were of diverse phylogenetic affiliation and were isolated from *Halichondria panicea* (HB strains) [46] and *Saccharina latissima* (synonym *Laminaria saccharina*) (LB strains) [47] collected at the Kiel Fjord, Germany, and also from different sponges collected from the Adriatic Sea near Rovinj, Croatia (AB strains). The strains belong to six different phylogenetic groups (Table 1). Additionally, type strains known to produce phenazines were used as positive controls: *Streptomyces cinnamonensis* DSM 1042^T^, *Pseudomonas chlororaphis* subsp. *chlororaphis* DSM 50083^T^, *Pseudomonas chlororaphis* subsp. *aureofaciens* DSM 6698^T^, and *Pseudomonas chlororaphis* subsp. *aurantiaca* DSM 19603^T^. For *S. cinnamonensis* DSM 1042^T^ phenazine gene sequences and the production of different endophenazines and PCA (phenazine-1-carboxylic acid) have been demonstrated [15]. *P. chlororaphis* subsp. *chlororaphis* produced chlororaphin [48], *P. chlororaphis* subsp. *aureofaciens* and *P. chlororaphis* subsp. *aurantiaca* produces 2-hydroxy-phenazine [42] and phenazine-1-carboxylic acid [41], respectively.

For identification of the strains 16S rRNA gene sequence analyses were carried out according to Thiel *et al.* 2007 [49]. Comparison of the 16S rDNA sequences was performed using the EMBL nucleotide database available at the European Bioinformatics Institute homepage using the Basic Local Alignment Search Tool (nucleotide blast) [50] and the Ribosomal Database Project (RDP) database [51].

### 3.2. Design of Oligonucleotides for Molecular Detection of phzE Phenazine Gene Fragments

For the primer construction, amino acid sequences and nucleotide sequences of different *phz*E genes were retrieved from the European Bioinformatics Institute homepage and aligned using the program *CLUSTAL_X* [52]. Nucleotide sequences were deduced from amino acid sequences. The alignment was analyzed manually. The following *phz*E sequences were used for primer design: *Streptomyces cinnamonensis* (AM384985/CAL34110/68793…70757; putative 2-amino-2-desoxy-isochorismate synthase), *Pseudomonas chlororaphis* PCL1391 (AF195615/AAF17499/4873…6786; phenazine-1-carboxamide), *Pseudomonas aeruginosa* PAO1 (AF005404/AAC64488/3294…5177; pyocyanin), and *Pseudomonas aeruginosa* PAO1 (AE004091/AAG07601/4716660…4718543/AAG05292/2073555…2075438; phenazine biosynthesis protein PhzE). Primers (Table 4) were synthesized by MWG (Ebersbach, Germany). In order to check the specificity of the primers, the sequences of *phz*Ef and *phz*Er were compared with sequences from the EMBL database using the Basic Local Alignment Search Tool (http://blast.ncbi.nlm.nih.gov/Blast.cgi). This comparison revealed 100% identity of the primers with corresponding sites of phenazine biosynthesis genes. Since Ashenafi *et al*. (2008) [53] reported that the anthranilate synthase (SvTrpEG) of *Streptomyces venezuelae* has a high degree of amino acid sequence similarity to the phenazine biosynthetic enzyme PhzE, the corresponding nucleotide sequence (AF01267) was compared with the *phz*E primers using the bl2seq tool (http://blast.ncbi.nlm.nih.gov/Blast.cgi). No significant similarity was found indicating that false positive results are excluded.

### 3.3. Amplification and Identification of the Phenazine Gene Fragments

The amplification reactions were carried out in a final volume of 25 μL. Taq DNA Polymerase (New England BioLabs, Ipswich, UK; MA, 5 U reaction^−1^) with the ThermoPol Buffer Kit (New England BioLabs, Ipswich, UK; MA, USA) was applied. Primers *phz*Ef and *phz*Er were deployed in a 10 μM concentration. 1 μL of a preparation containing each deoxynucleoside triphosphate at a concentration of 2.5 mM was used. 10 to 50 ng DNA of all strains used in this study was employed as template.

The amplification of the *phz*F gene sequence of the pseudomonads used in this study was performed using puReTaq Ready-To-Go polymerase chain reaction Beads (Amersham Biosciences, Uppsala, Sweden) with the primers Ps_up1 and Ps_low1 [21]. Cycler conditions for both PCR experiments were as follows: Initial denaturation: 94 °C for 120 s followed by 36 cycles of primer annealing at 54.7 °C *(ph*zE) and 57 °C (*phz*F), respectively, for 60 s; primer extension at 72 °C for 120 s and denaturation at 94 °C for 60 s. A final extension of 72 °C for 420 s was performed. All PCR reactions were conducted in a T1 thermocycler (Whatman Biometra^®^, Göttingen, Germany). Results of the amplifications were checked on a 1.5% agarose gel stained with ethidium bromide. DNA sequencing was done according to Wiese *et al*. [47]. The comparison of the *phz*E and *phz*F fragments, respectively, was done in the EMBL nucleotide database available at the European Bioinformatics Institute homepage using the Basic Local Alignment Search Tool (blastx) [50].

### 3.4. Cultivation of phz*E* Strains

#### 3.4.1. Cultivation of *phz*E Positive Strain

All strains with a positive result for phenazine gene fragments were cultivated for subsequent chemical analysis of their cell extracts. They were grown on GYM agar plate (4 g glucose, 4 g yeast extract, 4 g malt extract, 2 g CaCO_3_, 15 g agar, 1 L water, pH 7.2) or MB agar plate (37.4 g Difco marine broth, 15 g agar, 1 L water, pH 7.2) at 28 °C for 17 days as well as in 100 mL and 1000 mL GYM (4 g glucose, 4 g yeast extract, 4 g malt extract, 1 L water, pH 7.2) or MB medium (37.4 g Difco marine broth, 1 L water, pH 7.2) at 28 °C and 120 rpm for seven days. The three *P. chlororaphis* strains were cultivated in 1000 mL King B medium [56] at 28 °C and 120 rpm for 24 h.

#### 3.4.2. Cultivation of *phz*E Negative Strains

All strains with a negative result for phenazine gene fragments were cultivated for subsequent chemical analysis of their cell extracts. The precultures were grown on TSB medium agar plates (tryptic soy broth[Difco], 12 g/L; NaCl 20 g/L; agar 15 g/L) at 28 °C for 1 day. A 1 cm^2^ piece of the agar plate was used for inoculation of the main culture. Main cultures were grown in 100 mL TSB medium (with four baffles) and KingB medium [56] at 28 °C and 120 rpm for one, three and six days.

### 3.5. Culture Extracts of phzE Positive and Negative Strains

For 1 L cultures the supernatants were separated from the cell mass pellets by centrifugation at 4.700 × g for 20 min and extracted separately. Cells were homogenized by addition of 150 mL 96% EtOH and using Ultra-Turrax (IKA, Staufen, Germany) at 13,000 rpm for 30 s. The extracts were dried *in vacuo* and redissolved in MeOH for further analyses. Supernatants and the other cultures were extracted with EtOAc by homogenization with the help of Ultra-Turrax at 16,000 rpm for 30 s, also dried *in vacuo* and redissolved in MeOH for further analyses.

### 3.6. Chemical Analysis of phzE Positive and Negative Strains

Reversed phase HPLC experiments were performed using a C_18_ column (Phenomenex Onyx Monolithic C18, 100 × 3.00 mm) applying an H_2_O (A)/MeCN (B) gradient with 0.1% HCOOH added to both solvents (gradient 0 min 5% B, 4 min 60% B, 6 min 100% B; flow 2 mL/min) on a VWR Hitachi Elite LaChrom system coupled to an ESI-ion trap detector (Esquire 4000, Bruker Daltonics). Dereplication of substances was realized by comparison of MS and UV data obtained by HPLC-UV/MS analyses used data from the Antibase [57] and the Chapman & Hall/CRC Dictionary of Natural Products databases [58]. For endophenazines A and B, 2-hydroxy-phenazine and phenazine-1-carboxylic acid structure was confirmed by ^1^H NMR analysis.

### 3.7. Nucleotide Sequence Accession Numbers

The nucleotide sequence data reported in the present study were deposited in the GenBank nucleotide sequence database under the accession numbers HM460698 (AB049), HM460699 (AB108), AJ849545 (YIM 90018), AM231308 (YIM 36723), GQ863906 (HB117), GQ863907 (HB122), GQ863918 (HB202), GQ863921 (HB253), GQ863922 (HB254), GQ863926 (HB291), AM749667 (LB066), AM913982 (LB114), AM913952 (LB129), AM913970 (LB150) and AM913971 (LB151) for 16S rRNA and HM460700-HM460715 for *phz*E gene fragments.

## 4. Conclusions

In conclusion, the application of the *phz*E primer system is a useful tool to indicate the presence of the phenazine biosynthetic pathway in various groups of bacteria. The proof of concept was shown for well known producers of phenazines, but also for marine *Streptomyces* sp. strain. This approach is particularly relevant, because many marine *Actinobacteria* turned out to be active phenazine producers and *Streptomyces* strains are known to synthesize phenazines with anticancer and/or anti-infective activities [59]. The method used in this study offers a promising method to test the ability of producing phenazines in new isolates of all kinds of bacteria including marine *Actinobacteria.*

## Figures and Tables

**Table 1.** Distribution of *phz*E phenazine genes among major phylogenetic groups and phenazine production of cultures (number of strains).

**Phylogenetic group**	**Number of strains**		
	**Analyzed**	**PCR amplification *phz*E gene positive**	**Producing phenazines in culture**
*Actinobacteria*^a^	76	11	11
*Bacteroidetes*	2	0	0
*Firmicutes*	28	1	0
*Alphaproteobacteria*	21	2	0
*Betaproteobacteria*	2	0	0
*Gammaproteobacteria*^b^	39	3	3
**In total**	**168**	**17**	**14**

a including control strain *S. cinnamonensis.*

b including 3 control *P. chlororaphis* strains.

**Table 2.** Presence of phenazine biosynthesis genes in the strains investigated. Information on related type strains (according to 16S rRNA sequences) and sequence similarity of *phz*E genes to known phenazine genes is displayed. Length, similarity and original bearer of the genetic information of the *phz*E PCR products are also included.

**Strain no.**	**Next relative type strain and acc.-no.***^a^*	**Phylum***^b^*	**Sequence length***^c^*	**Related phenazine gene, acc.-no.***^d^***, similarity and producer**
Strains used as DSM 1042^T^	Positive control *Streptomyces cinnamonensis* DSM 1042^T^; DQ462657	A	127	*phz*E; CAL34110; 100%; *S. cinnamonenis*
DSM 6698^T^	*Pseudomonas chlororaphis* subsp. *aureofaciens* DSM 6698^T^; AY509898	GP	139	*phz*E: ADP21173; 100% *P. chlororaphis* *phz*F: ADP21174; 100% *P. chlororaphis*
DSM 19603^T^	*Pseudomonas chlororaphis* subsp. *aurantiaca* DSM 19603^T^; DQ682655	GP	137	*phz*E: ADP21173; 98% *P. chlororaphis* *phz*F: ADP21174 49% *P. chlororaphis*
DSM 50083^T^	*Pseudomonas chlororaphis* subsp. *chlororaphis* DSM 50083^T^; Z76673	GP	125	*phz*E; AAF17499; 92% *P. chlororaphis* *phz*F: AAF17500; 99% *P. chlororaphis*
Environmental	Isolates			
AB108	*Pseudovibrio ascidiaceicola* F423^T^; AB175663	AP	144	*phz*E; CAL34110; 95%; *S. cinnamonenis*
HB117	*Streptomyces fulvorobeus* LMG 19901^T^; AJ781331	A	141	*phz*E; AAF17499; 73%; *P. chlororaphis*
HB122	*Streptomyces luridiscabiei* S63^T^ AF361784	A	141	*phz*E; AAF17499; 74%; *P. chlororaphis*
HB202	*Streptomyces mediolani* LMG 20093^T^; AJ781354	A	91	*phz*E; NP_252903; 84%; *P. aeruginosa*
HB253	*Micromonospora matsumotoense* IMSNU 22003^T^; AF152109	A	144	*phz*B; AAF17496; 67%; *P. chlororaphis*
HB254	*Micromonospora matsumotoense* IMSNU 22003^T^; AF152109	A	140	*phz*E; AAF17499; 73%; *P. chlororaphis*
HB291	*Streptomyces fulvorobeus* LMG 19901^T^; AJ781331	A	140	*phz*E; AAF17499; 73%; *P. chlororaphis*
LB066	*Kiloniella laminariae* LD81^T^; AM749667	AL	132	*phz*E; CAL34110; 91%; *S. cinnamonenis*
LB114	*Streptomyces flavogriseus* DSM 40323^T^; AJ494864	A	141	*phz*E; AAF17499; 79%; *P. chlororaphis*
LB129	*Streptomyces fimicarius* ISP 5322^T^; AY999784	A	145	*phz*B; AAF17496; 75%; *P. chlororaphis*
LB150	*Streptomyces luridiscabiei* S63^T^; AF361784	A	132	*phz*B; AAF17496; 74%; *P. chlororaphis*
LB151	*Streptomyces griseus* ATCC51928^T^; AF112160	A	133	*phz*E; AAF17499; 65%; *P. chlororaphis*

*^a^* NCBI accession number. (all sequences were at least 98.5% similar to the corresponding type strain).

^b^ A = *Actinobacteria*, AP = *Alphaproteobacteria*, GP = *Gammaproteobacteria,* F = *Firmicutes*.

^c^ Given is the number of amino acids.

^d^ NCBI accession number.

**Table 3.** Known and putative novel phenazines from the strains studied.

**Strain no.**	**Next relative type strain**	**[M^+^]**	**UV absorption maxima (nm)***^a^*	**Dereplication of phenazines**
Strains used as DSM 1042^T^	positive control *Streptomyces cinnamonensis* DSM 1042^T^	206	327, 249, 212	no hit in database
		224	371, 249, 215	phenazine-1-carboxylic acid [41]
		268	375, 256, 223	phenazine-1,6-dicarboxylic acid [15]
		292	371, 254, 214	endophenazine A [15] *^c^*
		306	387, 269, 211	no hit in database
		308	372, 249, 212	endophenazine C [15]
		322	375, 256, 223	endophenazine B [15]*^c^*
		336	372, 249, 212	no hit in database
DSM 6698^T^	*Pseudomonas chlororaphis* subsp. *aureofaciens* DSM 6698^T^	196	368, 257, 219	2-hydroxy-phenazine [42]
		224	371, 249, 215	phenazine-1-carboxylic acid [41]
DSM 19603 ^T^	*Pseudomonas chlororaphis* subsp. *aurantiaca* DSM 19603^T^	196	368, 257, 219	2-hydroxy-phenazine [42]^c^
		224	371, 249, 215	phenazine-1-carboxylic acid [41]^c^
DSM 50083 ^T^	*Pseudomonas chlororaphis* subsp. *chlororaphis* DSM 50083^T^	223	370, 248, 213	chlororaphin [43]
Environmental	Isolates			
HB117	*Streptomyces fulvorobeus* LMG 19901^T^	494	370(br), 274, 224	Senacarcin A
		512	370(br), 275, 230	saphenyl ester D [29]
HB122	*Streptomyces luridiscabiei* S63^T^	492	376, 275, 235sh	saphenyl ester D [29]
		496	438sh, 383(br), 276, 227	no hit in database
		498	419sh, 393-325, 289, 253sh, 220	no hit in database
		508	376, 275, 235sh	no hit in database
		510	430(br), 325, 224	derivative of aestivophoenin C [44]
		512	432(br), 327, 226	aestivophoenin C [44]
HB202	*Streptomyces mediolani* LMG 20093^T^	396	368, 364sh, 351sh, 252, 218	streptophenazines E [39]*^c^*
		410	371, 364sh, 354sh, 252, 213	streptophenazines C [39]
		410	368, 364sh, 351sh. 252, 218	streptophenazines D [39]*^c^*
		424	367, 363sh, 350sh, 252, 215	streptophenazines A [39]
		424	368, 364sh, 351sh, 252, 218	streptophenazines B [39]*^c^*
		438	368, 364sh, 353sh, 252, 215	streptophenazines F [39]*^c^*
		438	368, 363sh, 351sh, 252, 214	streptophenazines G [39]
		440	368, 363sh, 352sh, 252, 215	streptophenazines H [39]
HB253	*Micromonospora matsumotoense* IMSNU 22003^T^	260	458, 302sh, 261, 232	no hit in database
		465	362sh, 345, 299, 221	no hit in database
		566	362sh, 345, 299, 221	no hit in database
HB254	*Micromonospora matsumotoense* IMSNU 22003^T^	451	361, 343, 352, 301, 223	no hit in database
HB291	*Streptomyces fulvorobeus* LMG 19901^T^	492	376, 275, 235sh	saphenyl ester D [29]
		496	438sh, 383(br), 276, 227	no hit in database
		498	419sh, 393-325, 289, 253sh, 220	no hit in database
		508	376, 275, 235sh	no hit in database
		510	430(br), 325, 224	derivative of aestivophoenin C [44]
		512	432(br), 327, 226	aestivophoenin C [44]
LB114	*Streptomyces flavogriseus* DSM 40323^T^	n.d.*^b^*	370, 270, 244	no hit in database
		n.d.*^b^*	419, 367, 305, 228	no hit in database
LB129	*Streptomyces fimicarius* ISP 5322^T^	296	366, 249, 214	phencomycin methyl ester [9]
		238	366, 249, 214	1-carboxymethyl phenazine
LB150	*Streptomyces luridiscabiei* S63^T^	510	sh401, 378, 274, 227	no hit in database
LB151	*Streptomyces griseus* ATCC 51928^T^	510	sh401, 378, 274, 227	no hit in database

^a^ sh, shoulder, br, broad.

^b^ n.d., not detectable.

^c^ substance was isolated by prepHPLC and structure was identified by UV-MS and ^1^H NMR analysis (data not shown).

**Table 4.** Primers used in this study.

**Primer**	**Sequence**	**Function**	**Ref.**
27f	5′-GAGTTTGATCCTGGCTCAG-3′	PCR of the 16S rRNA gene	[54]
1492r	5′-GGTTACCTTGTTACGACTT-3′	PCR of the 16S rRNA gene	[54]
534r	5′-ATTACCGCGGCTGCTGG-3′	Sequencing of the 16S rRNA gene	[55]
342f	5′-TACGGGAGGCAGCAG-3′	sequencing of the 16S rRNA gene	[55]
790f	5′-GATACCCTGGTAGTCC-3′	sequencing of the 16S rRNA gene	[50]
*phz*Ef	5′-GAAGGCGCCAACTTCGTYATCAA-3′	PCR and sequencing of *phz*E gene	this study
*phz*Er	5′-GCCYTCGATGAAGTACTCGGTGTG-3′	PCR and sequencing of *phz*E gene	this study
Ps_up1	5′-ATCTTCACCCCGGTCAACG-3′	PCR and sequencing of *phz*F gene	[21]
Ps_low1	5′-CCRTAGGCCGGTGAGAAC-3′	PCR and sequencing of *phz*F gene	[21]
